# Effects of linear and daily undulating periodized resistance training programs on measures of muscle hypertrophy: a systematic review and meta-analysis

**DOI:** 10.7717/peerj.3695

**Published:** 2017-08-22

**Authors:** Jozo Grgic, Pavle Mikulic, Hrvoje Podnar, Zeljko Pedisic

**Affiliations:** 1 Institute of Sport, Exercise and Active Living (ISEAL), Victoria University, Melbourne, Australia; 2 University of Zagreb, Faculty of Kinesiology, Zagreb, Croatia

**Keywords:** Skeletal muscle, Cross-sectional area, Lean body mass

## Abstract

**Background:**

Periodization is an important component of resistance training programs. It is meant to improve adherence to the training regimen, allow for constant progression, help in avoiding plateaus, and reduce occurrence and severity of injuries. Previous findings regarding the effects of different periodization models on measures of muscle hypertrophy are equivocal. To provide a more in-depth look at the topic, we undertook a systematic review of the literature and a meta-analysis of intervention trials comparing the effects of linear periodization (LP) and daily undulating periodization (DUP) resistance training programs on muscle hypertrophy.

**Materials and Methods:**

A comprehensive literature search was conducted through PubMed/MEDLINE, Scopus, Web of Science, SPORTDiscus, Networked Digital Library of Theses and Dissertations (NDLTD) and Open Access Theses and Dissertations (OATD).

**Results:**

The pooled standardized mean difference (Cohen’s d) from 13 eligible studies for the difference between the periodization models on muscle hypertrophy was −0.02 (95% confidence interval [−0.25, 0.21], *p* = 0.848).

**Conclusions:**

The meta-analysis comparing LP and DUP indicated that the effects of the two periodization models on muscle hypertrophy are likely to be similar. However, more research is needed in this area, particularly among trained individuals and clinical populations. Future studies may benefit from using instruments that are more sensitive for detecting changes in muscle mass, such as ultrasound or magnetic resonance imaging.

## Introduction

Regular participation in resistance training has been recommended to adults seeking general health and fitness by the World Health Organization (2010), US Department of Health and Human Services (2008), American College of Sports Medicine (2009) and many other global public health authorities. Resistance training is recommended as it may improve muscle mass, strength, and bone mass (Petersen, Hastings & Gottschall, 2017). It may also be associated with greater mobility (Yamamoto et al., 2016), increased health-related quality of life (Goldfield et al., 2017), increased resting metabolic rate (Campbell et al., 1994), and reduced risk of type 2 diabetes (Grøntved et al., 2012). Population-based studies from Australia and the US have found that 18.6% and 31.7% of adults, respectively, participate in muscle-strengthening physical activities at least two times a week (Bennie et al., 2016; Loustalot et al., 2013). Muscle mass is also thought to be an important factor influencing performance in many sports disciplines, particularly in physique sports (e.g., bodybuilding, men’s physique, etc.). Resistance training designed to promote muscle hypertrophy is, therefore, for many athletes an important part of their training regimen.

According to Haff & Triplett (2016), periodization enables systematic, sequential, and integrative scheduling and programming of training sessions to maximize specific physiological adaptations underpinning performance outcomes. Commonly used forms of periodization are the linear or classic periodization model (LP) and the nonlinear or undulating periodization model. According to Rhea and colleagues (Rhea et al., 2002), LP gradually increases training intensity and decreases volume, with these changes being made approximately every four weeks. A nonlinear form of periodization was previously described by Poliquin (1988) and is characterized by more frequent alterations in intensity and volume. The undulating periodization models can be performed on a weekly or daily basis. Daily fluctuations in intensity and load are often referred to as “the daily undulating periodization” (DUP), while the periodization with weekly fluctuations is termed “the weekly undulating periodization” (WUP).

The current empirical evidence provides insights regarding training frequency (Schoenfeld, Ogborn & Krieger, 2016), volume (Schoenfeld, Ogborn & Krieger, 2017), rest intervals (Schoenfeld et al., 2016b), and repetition ranges (Schoenfeld et al., 2016a). Despite these evidence-based general suggestions for designing a training protocol for muscle hypertrophy, there is a paucity of evidence regarding different periodization strategies.

It seems that most resistance training programs utilize some form of periodization, but it is still unclear whether the effectiveness of periodization is mainly related to the principle of specificity when analyzing strength (Mattocks et al., 2016), or more determined by the varying structure of the training programs and the differences in the volume of training (Kok, Hamer & Bishop, 2009). A recent systematic review and meta-analysis by Harries, Lubans & Callister (2015) found no significant differences between LP and undulating periodization models in increasing upper- and lower-body strength. However, the study did not analyze whether the two types of periodization differ in their effects on muscle hypertrophy. Findings about the effects of these two forms of periodization in volume equated comparisons on muscle hypertrophy seem to be equivocal, with some studies indicating greater benefits from the DUP model (Simão et al., 2012; Spineti et al., 2014) and others demonstrating no significant differences (De Lima et al., 2012; Prestes et al., 2015).

To provide a better understanding of the current evidence regarding the LP and DUP models and their effects on measures of muscle hypertrophy, we conducted a systematic review of the literature coupled with a meta-analysis. A more informed understanding of the topic may be of benefit to personal trainers and strength and conditioning coaches, professional athletes competing in many sports, and millions of those participating in muscle-strengthening activities for recreational and/or health purposes (Bennie et al., 2016; Centers for Disease Control and Prevention, 2013; Harada et al., 2008; Loustalot et al., 2013; Schoenborn, Adams & Peregoy, 2013). The focus of this paper was on LP and undulating periodization, specifically, DUP, as these models are most commonly used in intervention studies examining the impact of periodization on muscular adaptations (Harries, Lubans & Callister, 2015).

## Materials and Methods

The methods were specified in advance in the PROSPERO International prospective register of systematic reviews (ref: CRD42016047795).

### Search strategy

A systematic literature search was conducted according to the guidelines for Preferred Reporting Items for Systematic Reviews and Meta-Analyses (PRISMA) (Moher et al., 2009). A comprehensive search of the PubMed/MEDLINE, SCOPUS, Web of Science (including Science Citation Index Expanded, Social Sciences Citation Index, and Arts & Humanities Citation Index), SPORTDiscus, Networked Digital Library of Theses and Dissertations (NDLTD) and Open Access Theses and Dissertations (OATD) databases was conducted. In all databases, a search through titles, abstracts, and keywords of indexed documents was performed from the inception of indexing to September 14th, 2016, by combining the search terms for periodization (periodis* and periodiz*) with the following search terms: resistance training; muscle hypertrophy; strength training; muscle mass; skeletal muscle; cross-sectional area; body composition; strength exercise; strengthening exercise; resistance exercise; weight training; weight lifting; muscle strengthening; muscular strengthening; muscle training; muscle exercise; muscle toning; muscle gain; muscle volume; weight bearing exercise; weight bearing training; bodybuilding; body building. The full syntax with Boolean operators used for the literature search may be found in Appendix S1. As part of the secondary search, we also screened the reference lists of all selected documents and the studies that have cited the included studies.

### Inclusion criteria

Studies were assessed for eligibility based on the following inclusion criteria: (a) an experimental trial published in an English-language peer-reviewed journal or a doctoral thesis; (b) it compared the use of a LP resistance training program to a DUP resistance training program or to a mixture of WUP and DUP resistance training program in a traditional dynamic resistance exercise using coupled concentric and eccentric muscle actions; (c) the study used at least one method of estimating changes in muscle mass (studies using only measures of circumference without controlling for body fat changes were excluded as they do not provide an accurate assessment of changes in lean body mass); (d) the volume was equated between the different periodization models; (e) the study lasted for a minimum of six weeks and involved participants with no musculoskeletal injury or any other health condition that could directly or through medications associated with the management of a given condition be expected to impact the hypertrophic responseto resistance exercise (i.e., coronary artery disease and angiotensin receptor blockers), as done in previous research examining muscle hypertrophy (reviews Schoenfeld, Ogborn & Krieger, 2017).

### Study selection

The study selection was conducted independently by two authors (JG and HP) in order to minimize potential selection bias. Disagreements between the investigators were resolved through discussion and mutual consensus, and any remaining disagreements were settled by the third investigator (PM). Studies were excluded based on their title or, where necessary, by reading the abstract or full text. Attempts were made to contact authors to provide any additional data needed for a given study to meet the inclusion criteria.

### Study coding and data extraction

The studies meeting the inclusion criteria were read and independently coded by two investigators (JG and HP) using a predefined spreadsheet. The following data was extracted: (a) descriptive information of participants by gender, age (ages 10–18 years were classified as adolescents, aged 19–39 classified as young adults, aged 40–64 classified as middle-aged adults, aged ≥65 classified as elderly), and experience in resistance training (classified as untrained if they had <1 year of experience and as trained if they had ≥1 year of experience); (b) the number of participants in each group; (c) the type of study design; (d) the duration of the intervention trial; (e) the characteristics of both resistance training models; (f) the type of morphologic measurements (skinfolds, circumferences, ultrasound, magnetic resonance imaging—MRI, dual energy X-ray absorptiometry—DXA, air displacement plethysmography— BOD-POD, and/or bio-impedance analysis—BIA); (g) the region of the body measured for the studies that used skinfolds, circumferences, ultrasound or MRI; and (h) main findings related to muscle hypertrophy. Coding was crosschecked between the coders, and all discrepancies were resolved through discussion. The assessment of potential coder drift was performed by randomly selecting 30% of the studies for recoding, as described by Cooper, Hedges & Valentine (2009).

### Quality assessment

To assess the methodological quality of the studies, we used the PEDro Scale (Maher et al., 2003). The PEDro scale is a 11-criteria checklist for assessing the methodological quality of randomized controlled trials. Studies are awarded 0–10 points, depending on the number of criteria they meet (the first item is not used to calculate the summary score). Due to the nature of exercise interventions, no included studies could theoretically do blinding of subjects and investigators, and hence none could earn points on the PEDro Scale items 5 and 6. In addition to presenting the overall PEDro Scale score, we therefore also presented the percentage of the maximal possible score, which is 8 points. Details about the scale items can be found elsewhere (Maher et al., 2003). The same authors that performed the search and coding of the studies independently completed the PEDro Scale. Any disagreement was resolved by mutual consensus or by consensus with a third investigator (PM).

### Statistical analysis

Differences in the pre-post data for each individual study and measured muscle group were calculated as a percent change as follows:

\begin{eqnarray*}(\text{Post-training measure}-\text{Pre-training measure})/\text{Pre-training measure}\times 100. \end{eqnarray*}

A meta-analysis was performed using the “Comprehensive Meta-analysis” software (Biostat Inc., Englewood, NJ, USA). The data entry format that was used for the calculation of standardized mean differences (Cohen’s d [SMD]) and its 95% confidence interval (CI) was “means, standard deviation (SD) pre and post, sample size (*n*) in each group, pre/post correlation”. As none of the studies reported correlation and it could not be estimated with high precision (Borenstein et al., 2009) the data was standardized by post score SD values. When the data is standardized by post score SD, the correlation values are not needed for the calculation of SMDs. Unlike the standardization by pre-intervention SD values, this method provides more insights regarding the variability of the training effect (Dankel et al., 2017c). In LP vs. DUP comparisons, where the data showed a greater increase from pre to post values for the LP group, this was expressed as the positive effect direction, and where the data showed a greater increase from pre to post values for the DUP group, this was expressed as the negative effect size direction. The study by Spineti et al. (2014) had two LP and DUP groups, so we analyzed their results separately. Four meta-analyses were performed: (i) including all 13 studies; (ii) a subgroup analysis including the studies that used direct measures of muscle hypertrophy; (iii) a subgroup analysis including the studies that used indirect measures of muscle hypertrophy; and (iv) a subgroup analysis including the studies with training periods longer than 11 weeks. Both measures were considered as muscle hypertrophy, assuming that changes in lean body mass with resistance training are mainly attributed to gains in muscle tissue. The random effect model was used for performing the meta-analysis. Sensitivity analysis was performed by (a) using the same software by removing one study at a time and then examining the outcomes and (b) removing both of the studies (Foschini et al., 2010; Inoue et al., 2015) that combined resistance training with aerobic training. The statistical significance threshold was set at *p* < 0.05.

## Results

**Figure 1:**
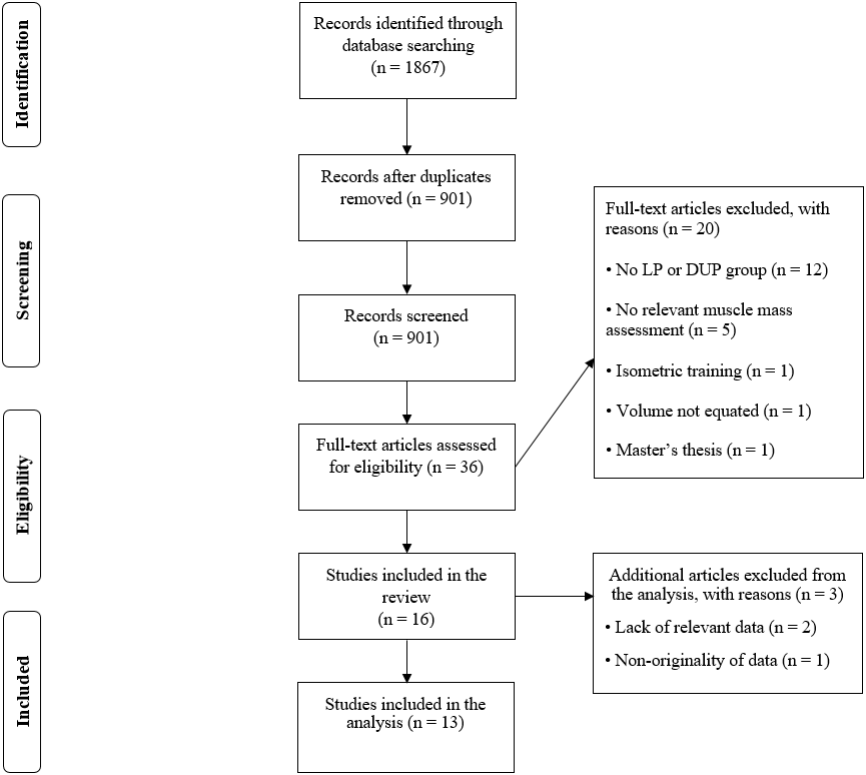
Flow diagram of the search and study selection process.

A total of 1,867 records were found in the primary search (Fig. 1, Supplemental Information). A total of 36 papers were read in full to assess their eligibility for inclusion. A total of 16 studies were found to have potential to meet all the inclusion criteria. The authors of 4 studies (Ahmadizad et al., 2014; Prestes et al., 2009b; Rhea et al., 2002; Spineti et al., 2013) were asked to provide additional data required for their study to meet the inclusion criteria. For two studies (Prestes et al., 2009b; Rhea et al., 2002) the additional data was not provided upon our request, and they were, therefore, not included in the review. A study (Spineti et al., 2013) was removed due to non-originality of the presented data. The final number of studies included in this review was thirteen, with five studies using direct measures of muscle hypertrophy and eight studies using indirect measures of muscle hypertrophy. All studies included in the systematic review received ethics approval from the local Institutional Review Board.

The pooled sample size from all the included studies was *n* = 417, with 303 participants involved in either LP or DUP resistance training program. The average (±SD) duration of the studies was 12 ± 5 weeks. Most studies were conducted on untrained populations with little or no resistance training experience (Ahmadizad et al., 2014; De Lima et al., 2012; Foschini et al., 2010; Harries, Lubans & Callister, 2016; Inoue et al., 2015; Kok, Hamer & Bishop, 2009; Kok, 2006; Prestes et al., 2015; Simão et al., 2012; Souza et al., 2014; Spineti et al., 2014) with only one study (Monteiro et al., 2009) comparing the effects of different periodization models on trained individuals.

**Table 1:** Studies on the difference between the effects of LP and DUP periodization models on muscular hypertrophy: summary of findings and assessment of methodological quality.

Study	Publication type	Participants	Design	Study duration	Hypertrophy/ lean body mass measurement	Findings	Methodological quality^a^	Percentage of the theoretical maximum score for exercise intervention trials
Ahmadizad et al. (2014)	Published, peer reviewed	32 young untrained overweight men	Random assignment either to a control group (*n* = 8), or non-periodized (*n* = 8), a LP (*n* = 8) or a DUP (*n* = 8) resistance training program performing 6 different resistance training exercises. A mixture of both free weight and machine based multi-joint and single joint isolation exercises was used. The training was carried out 3 times per week.	8 weeks	BIA	No significant differences between groups in changes in lean body mass (1.9 % increase for the LP group, 2.0 % increase for the DUP group).	6	75%
Buford et al. (2007)	Published, peer reviewed	18 young untrained men and 10 young untrained women	Random assignment either to a LP (*n* = 9), a DUP (*n* = 10) or a WUP (*n* = 9) resistance training program performing 14 different resistance training exercises. A mixture of both free weight and machine based multi-joint and single-joint isolation exercises was used. The training was carried out 3 times per week.	9 weeks	Chest and thigh circumference (with 7 site skinfolds to control for body fat changes)	No significant differences between groups in changes in muscle girth (2.0% increase for the LP group, 0.2% increase for the DUP group in the chest circumference, 6.6% increase for the LP group, 3.7% increase for the DUP group in the thigh circumference). The LP group decreased body fat by 1.3% (5.0%), while the DUP group decreased body fat by 1.4% (6.6%).	6	75%
De Lima et al. (2012)	Published, peer reviewed	28 young untrained women	Random assignment either to a control group (*n* = 8), or a LP (*n* = 10) or a DUP (*n* = 10) resistance training program performing 16 different resistance training exercises. A mixture of both free weight and machine based multi-joint and single-joint isolation exercises was used. The training was carried out 4 times per week.	12 weeks	Triceps, suprailiac and thigh skinfolds	No significant differences between groups in changes in lean body mass (4.7% increase for the LP group, 3.5% increase for the DUP group).	6	75%
Foschini et al. (2010)	Published, peer reviewed	32 untrained obese adolescents (boys: *n* = 15, girls: *n* = 17)	Random assignment either to a LP (*n* = 16) or a DUP (*n* = 16) resistance training program performing 10 different resistance training exercises. A mixture of both free weight and machine based multi-joint and single-joint isolation exercises was used. Participants additionally performed 30 min of aerobic exercise. The training was carried out 3 times per week.	14 weeks	BOD-POD	No significant differences between groups in changes in lean body mass (3.4% increase for the LP group, 2.4% increase for the DUP group).	6	75%
Harries, Lubans & Callister (2016)	Published, peer reviewed	26 untrained adolescent boys	Assignment either to a control group (*n* = 10), or a LP (*n* = 8) or a DUP (*n* = 8) resistance training program performing 11 different resistance training exercises. A mixture of both free weight and machine based multi-joint and single-joint isolation exercises was used. The training was carried out 2 times per week.	12 weeks	BIA	No significant differences between groups in changes in lean body mass (2.1% decrease for the LP group, 1.0% increase for the DUP group).	7	87%
Inoue et al. (2015)	Published, peer reviewed	45 untrained obese adolescents (boys: *n* = 17, girls: *n* = 28)	Random assignment either to aerobic training, (*n* = 20), or a LP (*n* = 13) or a DUP (*n* = 12) resistance training program performing 10 different resistance training exercises. A mixture of both free weight and machine based multi-joint and single-joint isolation exercises was used. Participants in the LP and DUP groups additionally performed 30 min of aerobic exercise. The training was carried out 3 times per week.	26 weeks	BOD-POD	No significant differences between groups in changes in lean body mass (7.9% increase for the LP group, 2.6% increase for the DUP group).	5	62%
Kok (2006)	Doctoral dissertation	16 young untrained women	Random assignment based on the participants squat index (1RMSQ/mass) either to a LP (*n* = 8) or a DUP (*n* = 8) resistance training program performing 10 different resistance training exercises. A mixture of both free weight and machine based multi-joint and single-joint isolation exercises was used. The training was carried out 3 times per week.	12 weeks	Ultrasound performed on the right rectus femoris	No significant differences between groups in changes in muscle thickness (3.2% increase for the LP group, 12.9% increase for the DUP group in the cross sectional area).	5	62%
Kok, Hamer & Bishop (2009)	Published, peer reviewed	20 young untrained women	Random assignment based on the participants squat index (1RMSQ/mass) either to a LP (*n* = 10) or a DUP (*n* = 10) resistance training program performing 10 different resistance training exercises. A mixture of both free weight and machine based multi-joint and single-joint isolation exercises was used. The training was carried out 3 times per week.	12 weeks	Ultrasound performed on the right rectus femoris	No significant differences between groups in changes in muscle thickness (8.7% increase for the LP group, 14.8% increase for the DUP group in the cross sectional area)	6	75%
Monteiro et al. (2009)	Published, peer reviewed	27 young trained men	Random assignment either to a non-periodized (*n* = 9), a LP (*n* = 9) or a DUP (*n* = 9) resistance training program performing 15 different resistance training exercises. A mixture of both free weight and machine based multi-joint and single-joint isolation exercises was used. The training was carried out 4 times per week.	12 weeks	Biceps, triceps, subscapular, and suprailiac skinfolds	No significant differences between groups in changes in lean body mass (1.2% increase for the LP group, 0.3% increase for the DUP group).	5	62%
Prestes et al. (2015)	Published, peer reviewed	49 untrained elderly women	Random assignment either to a control group (*n* = 10), or a LP (*n* = 20) or a DUP (*n* = 19) resistance training program performing 10 different resistance training exercises. A mixture of both free weight and machine based multi-joint and single joint isolation exercises was used. The training was carried out 2 times per week.	16 weeks	DXA	No significant differences between groups in changes in lean body mass (1.6% decrease for the LP group, 1.7% increase for the DUP group).	5	62%
Simão et al. (2012)	Published, peer reviewed	30 young untrained men	Random assignment either to a control group (*n* = 9), a LP (*n* = 10) or a to a mixed WUP and DUP (*n* = 11) resistance training program performing 4 different resistance training exercises: barbell bench press, machine front lat-pull down, machine triceps extension, and the straight-bar standing biceps curl. The training was carried out 2 times per week.	12 weeks	Ultrasound performed on the right biceps and triceps	No significant differences between groups in changes in muscle thickness (5.7% increase for the LP group, 9.1% increase for the DUP group in the biceps, 0.8% increase for the LP group, 4.3% increase for the DUP group in the triceps).	6	75%
Souza et al. (2014)	Published, peer reviewed	31 young untrained men	Participants from each quartile according to their quadriceps cross-sectional were randomly assigned to a control group (*n* = 5), or a non-periodized (*n* = 9), a LP (*n* = 9) or a DUP (*n* = 8) resistance training program performing 2 different resistance training exercises for the lower body: squat and knee extensions. The training was carried out 2 times per week.	6 weeks	MRI performed on the dominant leg quadriceps	No significant differences between groups in changes in cross-sectional area (4.6% increase for the LP group, 5.2% increase for the DUP group).	6	75%
Spineti et al. (2014)	Published, peer reviewed	53 young untrained men	Random assignment either to a control group (*n* = 9), or a LP progressing from large to small (LG) muscle groups (*n* = 10), a LP progressing from small to large (SG) muscle groups (*n* = 13), a DUP progressing from large to small (LG) muscle groups (*n* = 11), a DUP progressing from small to large (SG) muscle groups (*n* = 10) resistance training program performing 4 different resistance training exercises: biceps curl, triceps extension, lat pull down and bench press. The training was carried out 2 times per week.	12 weeks	Ultrasound performed on the right biceps and triceps	No significant differences between groups in changes in muscle thickness (5.8% and 3.5% increase for the LP groups, 9.3% and 8.2% increase for the DUP groups in the biceps, 0.6% and 9.0% increase for the LP groups, 4.5% and 6.8% increase for the DUP groups in the triceps)	6	75%

**Notes.**

a Denotes the total score on the PEDro scale.

LP linear periodization DUP daily undulating periodization WUP weekly undulating periodization BIA bio-impedance analysis BOD-POD air displacement plethysmography DXA dual energy X-ray absorptiometry MRI magnetic resonance imaging

The changes in measures of muscle hypertrophy from pre- to post-training intervention are presented in Table 1. The unweighted mean percent change across studies was 3.9% ± 3.0% (95% CI [2.5, 5.3], (0.32% per week of training)) for the LP and 5.1% ± 4.2% (95% CI [3.1, 7.1], (0.43% per week of training)) for the DUP resistance training group. The mean percent changes for the subgroup of studies that used indirect measures of muscle hypertrophy were 3.5% ± 2.9% (95% CI [1.2, 5.0]) for LP and 1.9% ± 1.3% (95% CI [1.1, 2.8]) for DUP. The mean percent changes for the subgroup of studies that used direct measures of muscle hypertrophy were 4.7% ± 3.0% (95% CI [2.7, 6.6]) for LP and 8.3% ± 3.7% (95% CI [6.0, 10.7]) for DUP.

**Figure 2:**
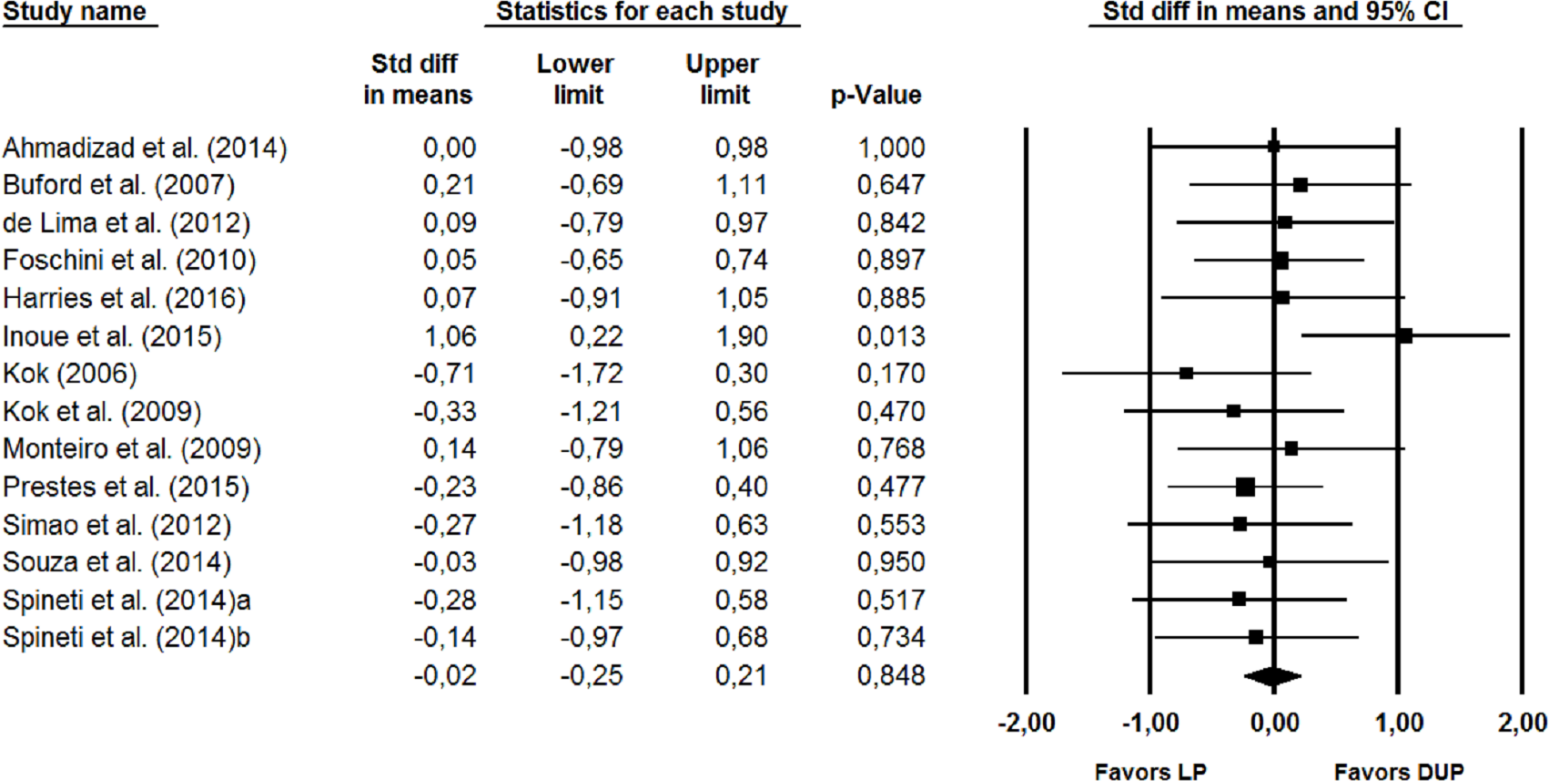
Forest plot showing differences between the effects plot of LP and DUP periodization models on measures of muscle hypertrophy. The size of the plotted squares reflects the relative statistical weight of each study. The numbers on the *X*-axis denote the standardized mean differences expressed as Cohen’s d. The horizontal lines denote 95% confidence intervals (CI) for the standardized mean differences. DUP, daily undulating periodization; LP, linear periodization; Std diff, standardized difference.

The meta-analysis using random-effects model showed no significant differences (*p* = 0.848) between the effects of the two periodization models and their effects on muscle hypertrophy. The pooled Cohen’s d (SMD) for the difference between the periodization models was −0.02 (95% CI [−0.25, 0.21], *p* = 0.848) (Fig. 2). None of the sensitivity analyses removing one study at a time showed a significant difference between the two periodization models. No significant difference (*p* = 0.314) was also observed when the sensitivity analysis was performed by removing two studies (Foschini et al., 2010; Inoue et al., 2015) that included aerobic exercise together with the resistance training.

**Figure 3:**
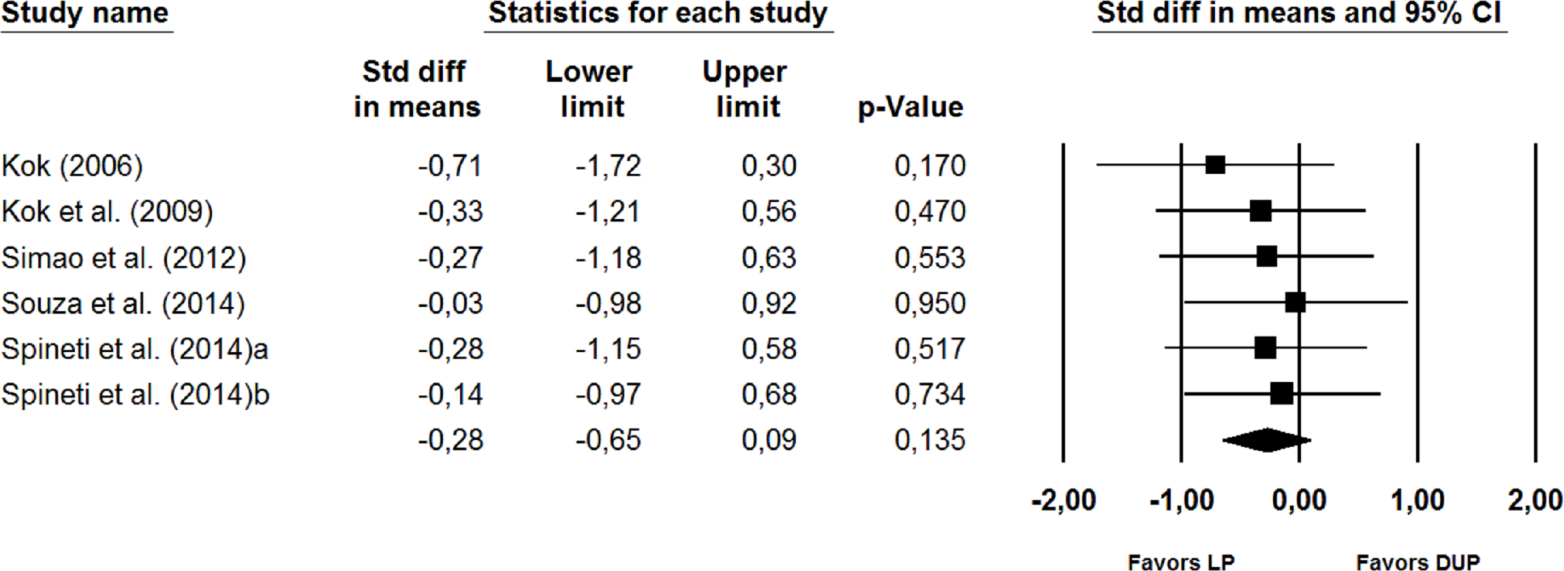
Forest plot showing differences between the effects plot of LP and DUP periodization models on measures of muscle hypertrophy (direct measures). The size of the plotted squares reflects the relative statistical weight of each study. The numbers on the *X*-axis denote the standardized mean differences expressed as Cohen’s d. The horizontal lines denote 95% confidence intervals (CI) for the standardized mean differences. DUP, daily undulating periodization; LP, linear periodization; Std diff, standardized difference.

**Figure 4:**
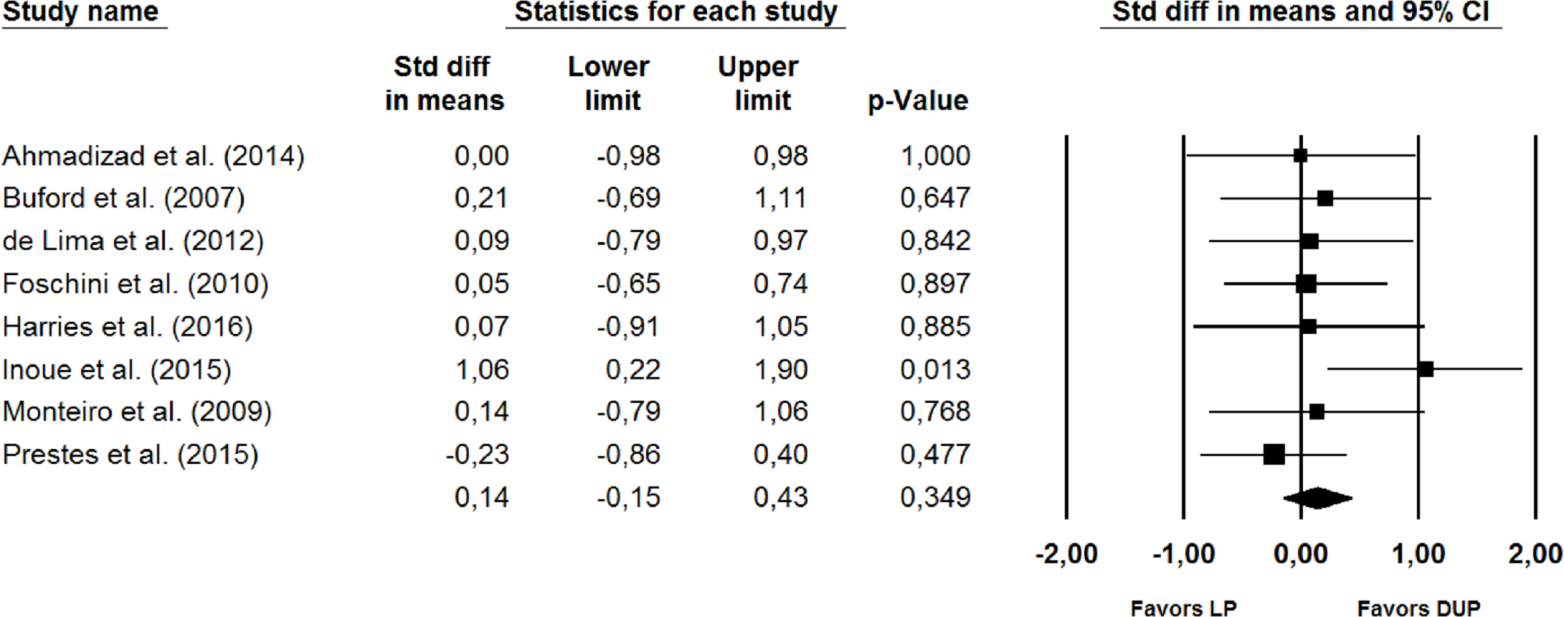
Forest plot showing differences between the effects plot of LP and DUP periodization models on measures of muscle hypertrophy (indirect measures). The size of the plotted squares reflects the relative statistical weight of each study. The numbers on the *X*-axis denote the standardized mean differences expressed as Cohen’s d. The horizontal lines denote 95% confidence intervals (CI) for the standardized mean differences. DUP, daily undulating periodization. LP, linear periodization. Std diff, standardized difference.

No significant differences between the two periodization models were observed in the subgroup analysis (Fig. 3) for studies that used direct measures of muscle hypertrophy (SMD = −0.28 (95% CI [−0.65, 0.09]), *p* = 0.135) for the studies that used indirect measures of muscle hypertrophy (SMD = 0.14 (95% CI [−0.15, 0.43]), *p* = 0.349) (Fig. 4), or for the studies that lasted >11 weeks (SMD = −0.41 (95% CI [−0.29, 0.21]), *p* = 0.747).

The PEDro scale quality assessment scores ranged from 5 to 7 points (Table 1) indicating moderate to high methodological quality across the included studies. The percentage of the theoretical maximum score for exercise intervention trials ranged from 62% to 87%.

## Discussion

This is the first systematic review to compare the effects of the LP and DUP resistance training programs on muscle hypertrophy. The meta-analysis comparing the two models on muscle hypertrophy did not indicate a significant difference between their effects. Therefore, the main finding of our review is that, when LP and DUP are volume-equated, there is no evidence to conclude that one type of periodization outperforms the other in the above-mentioned variables.

In the reviewed studies, the volume of training was equated between the LP and DUP experimental groups. It has been purported that appropriate training variable manipulation (i.e., periodization) is more important for producing gains than the total training volume (O’Bryant, Byrd & Stone, 1988). However, our meta-analysis does not corroborate this hypothesis. It might be that, for muscle gain, training volume is more important than the use of LP or DUP models (Kok, Hamer & Bishop, 2009), but it remains unclear whether these findings are generalizable to other forms of periodization (i.e., block periodization, reverse linear periodization etc.). The study by Kok, Hamer & Bishop (2009) showed that changes in strength and hypertrophy mirrored the training volume load, both in the LP and DUP groups. However, this may not always be the case, as it seems that even simply practicing the the strength assessment procedure may result in strength increases (Mattocks et al., 2017). Nevertheless, the effects of training volume on gains in muscle mass were confirmed in meta-analyses by Krieger (2010), and later by Schoenfeld and colleagues (2017). There seems to be a dose–response relationship between resistance training volume and increases in muscle mass, at least up to a certain level (Schoenfeld, Ogborn & Krieger, 2017). Although we observed no significant differences between LP and DUP in their effects on muscle hypertrophy, the study by Monteiro et al. (2009) found that the group that underwent a non-periodized resistance training lost 2.1 kg of lean body mass, while the LP and DUP groups increased lean body mass by 0.8 kg and 0.2 kg, respectively. Monteiro et al. (2009) suggest that both LP and DUP models might provide better results than a non-periodized approach. However, in the study by Monteiro et al. (2009), the absolute load was not adjusted throughout the duration of the study. It seems that not adjusting for increases in strength during the implementation of a resistance training program might hinder muscular adaptations. By contrast, the studies by Souza et al. (2014) and Ahmadizad et al. (2014) showed similar gains across the DUP, LP and the non-periodized groups. Furthermore, it seems that there is a general lack of evidence about effects of periodization in resistance training (Mattocks et al., 2016).

The DUP model involves increases and decreases in both volume and intensity throughout the training cycles (Rhea et al., 2003). In contrast, the traditional LP approach progresses from lower load/higher volume to heavier load/lower volume through the mesocycles. The latter pattern may not be ideal from a hypertrophy standpoint, as it has been suggested that muscle mass is not easily maintained through the low volume phases (Poliquin, 1988); however, as more recently highlighted by Bickel, Cross & Bamman (2011), this might depend on the age of the participants. It may be assumed that a reverse LP approach would be preferable for hypertrophic effects. In the reverse LP model, the high volume (i.e., hypertrophy) mesocycle is at the end of the macrocycle. Prestes et al. (2009a) compared traditional LP and reversed LP models in a group of resistance-trained men. Their study found greater increases in lean body mass in the traditional LP group. However, a limitation of the study is the use of skinfold thickness measures to assess changes in muscle mass. No previous studies using direct measures of muscle hypertrophy have compared the effects of DUP and the reverse LP resistance training models on muscle mass. This may be an interesting area to explore in future studies.

Our findings mainly relate to young individuals, with only one study (Prestes et al., 2015) that examined the effects of LP and DUP in a cohort of elderly woman. However, as most other studies, Prestes et al. (2015) observed no significant differences in the effects of LP and DUP on lean body mass. An important and commonly neglected factor when choosing a periodization model pertains to motivational factors. LP resistance training programs might provide a greater benefit to the individuals who favor tracking progression on a week-to-week or a month-to-month basis. By contrast, the DUP model might be more beneficial to the individuals who need more variety in order to adhere to the training programs. From the public health perspective, i.e., in relation to population-wide participation in resistance training, it might be worthwhile exploring these assumptions in future studies.

All but one reviewed study included untrained participants, which restricts generalization of the findings to the population of trained individuals. This is important to emphasize because it has been shown that training experience attenuates muscle protein synthesis (Kim, Staron & Phillips, 2005; Phillips et al., 1999; Tang et al., 2008) as well as intracellular anabolic signaling (Coffey et al., 2006; Gonzalez et al., 2015; Nader et al., 2014), resulting in slower hypertrophy adaptations in trained individuals when compared to untrained individuals. This may be a possible explanation of the overall non-significant difference between the effects of LP and DUP on muscle hypertrophy. However, as the recent study by Morton et al. (2016) has shown, even trained individuals may achieve robust gains in strength and muscle mass employing a non-periodized approach, if adequate progressive overload is used. Such findings may potentially put into question the need for implementing periodization in resistance training programs.

All included studies did report equating the training volume between the LP and DUP groups. However, a clearer standardization (i.e., training to volitional fatigue) has been suggested for studies examining muscle hypertrophy (Dankel et al., 2017a). Furthermore, some studies focused more on achieving muscular endurance (De Lima et al., 2012), while others focused more on strength and hypertrophy (Simão et al., 2012). Some studies examined the upper-body musculature (Simão et al., 2012; Spineti et al., 2014), while others examined the lower-body musculature (Souza et al., 2014). Additionally, some studies combined resistance training with aerobic exercise (Foschini et al., 2010; Inoue et al., 2015) and others (Simão et al., 2012; Spineti et al., 2014), combined DUP and WUP resistance training programs. Studies also used a range of different variants of LP and DUP periodization models. Also, changes in muscular hypertrophy were assessed using a range of methods and different muscle groups, with methods more sensitive to muscle changes, such as ultrasound and MRI (Bemben, 2002) used only in four out of thirteen reviewed studies. However, the variability of differences between the effects of LP and DUP was very low across the studies, with all but one effect size (Inoue et al., 2015) being classified as “trivial” and “small”. This indicates that the effects sizes are unlikely to have been significantly affected by the diverse methodological approaches used in the studies. Furthermore, based on the PEDro score, the methodological quality of most reviewed studies would be classified as moderate, regardless of whether the overall PEDro score or the maximum possible score for exercise intervention trials was used as the reference.

The average duration of the reviewed studies was 12 weeks, with only three studies lasting more than 12 weeks. It is, therefore, unclear whether the effects of periodization protocols would show larger differences over a longer term. It has been suggested that, to observe significant differences between periodization models, a full 1-year macrocycle might be needed (Buford et al., 2007). However, it might also be that both periodization models would result in similar changes in muscle mass even if applied throughout the whole macrocycle due to the ceiling effect with adaptation to resistance training (Dankel et al., 2017b). Considering the impediments for conducting long-term exercise interventions, it is not surprising that such studies are still lacking.

Further research in this field should aim to answer:

1. “Are there any differences between LP and DUP models in their effects on muscle hypertrophy among individuals with previous resistance training experience?”
2. “What are the effects of reverse LP models in comparison to DUP or traditional LP periodization schemes when assessing muscle hypertrophy with direct (i.e., ultrasound, MRI, CT) methods?”

On the point of Dankel et al. (2017c), it would be desirable for future studies to plot the individual responses to the intervention to increase the practical interpretability of the findings.

## Conclusions

The meta-analysis comparing LP and DUP approaches to resistance training indicated that the effects of the two periodization models on measures of muscle hypertrophy are likely to be similar. However, more research is needed in this area, particularly among trained individuals and clinical populations. Future studies may benefit from using instruments that are more sensitive for detecting changes in muscle mass, such as ultrasound or magnetic resonance imaging. Those interested in achieving maximal muscle hypertrophy should focus on training volume and progressive overload, while the use or the choice of a periodization model may be a matter of individual preference.

##  Supplemental Information

10.7717/peerj.3695/supp-1 Supplemental Information 1 PRISMA checklist

10.7717/peerj.3695/supp-2 Supplemental Information 2 Coding sheet

10.7717/peerj.3695/supp-3 Supplemental Information 3 Meta-analysis data (for the CMA software)

10.7717/peerj.3695/supp-4 Appendix S1 Syntax used for the search
