# Supplementary material for: Effects of linear and daily undulating periodized resistance training programs on measures of muscle hypertrophy: a systematic review and meta-analysis
Source: PeerJ. 2017 Aug 22;5:e3695. doi: 10.7717/peerj.3695 (PMC5571788; doi:10.7717/peerj.3695)
Supplement: Appendix S1 [file peerj-05-3695-s004.doc]

**Screening**

**Included**

**Eligibility**

**Identification**

Records identified through database searching
(n = 1867)

Records after duplicates removed
(n = 901)

Records screened
(n = 901)

Full-text articles assessed for eligibility
(n = 36)

Full-text articles excluded, with reasons (n = 20)

• No LP or DUP group (n = 12)

• No relevant muscle mass assessment (n = 5)

• Isometric training (n = 1)

• Volume not equated (n = 1)

• Master’s thesis (n = 1)

Studies included in the review (n = 16)

Studies included in the analysis (n = 13)

Additional articles excluded from the analysis, with reasons (n = 3)

• Lack of relevant data (n = 2)

• Non-originality of data (n = 1)
